# The Flavonoid Hesperidin Methyl Chalcone Targets Cytokines and Oxidative Stress to Reduce Diclofenac-Induced Acute Renal Injury: Contribution of the Nrf2 Redox-Sensitive Pathway

**DOI:** 10.3390/antiox11071261

**Published:** 2022-06-27

**Authors:** Allan J. C. Bussmann, Tiago H. Zaninelli, Telma Saraiva-Santos, Victor Fattori, Carla F. S. Guazelli, Mariana M. Bertozzi, Ketlem C. Andrade, Camila R. Ferraz, Doumit Camilios-Neto, Antônio M. B. Casella, Rubia Casagrande, Sergio M. Borghi, Waldiceu A. Verri

**Affiliations:** 1 Laboratory of Pain, Inflammation, Neuropathy, and Cancer, Department of Pathology, Londrina State University, Londrina 86057-970, Brazil; bussmann@uel.br (A.J.C.B.); tzaninelli@uel.br (T.H.Z.); telma.saraiva.santos@uel.br (T.S.-S.); vfattori@outlook.com (V.F.); carlafsg@yahoo.com.br (C.F.S.G.); marianambertozzi@gmail.com (M.M.B.); ketlemandrade94@gmail.com (K.C.A.); camila_ferraz96@hotmail.com (C.R.F.); 2 Department of Biochemistry and Biotechnology, Center of Exact Sciences, Londrina State University, Londrina 86057-970, Brazil; camiliosneto@uel.br; 3 Department of Internal Medicine, Center of Health Sciences, Londrina State University, Londrina 86039-440, Brazil; casella@uel.br; 4 Department of Pharmaceutical Sciences, Center of Health Sciences, Londrina State University, Londrina 86039-440, Brazil; rubiacasa@uel.br; 5 Center for Research in Health Sciences, University of Northern Paraná, Londrina 86041-140, Brazil

**Keywords:** citrus flavonoid, hesperidin methylchalcone, Nrf2, diclofenac, acute renal injury, oxidative stress

## Abstract

Hesperidin is derived from citrus fruits among other plants. Hesperidin was methylated to increase its solubility, generating hesperidin methyl chalcone (HMC), an emerging flavonoid that possess anti-inflammatory and antioxidant properties. The nuclear factor erythroid 2-related factor 2 (Nrf2) is a powerful regulator of cellular resistance to oxidant products. Previous data evidenced HMC can activate Nrf2 signaling, providing antioxidant protection against diverse pathological conditions. However, its effects on kidney damage caused by non-steroidal anti-inflammatory drugs (NSAIDs) have not been evaluated so far. Mice received a nephrotoxic dose of diclofenac (200 mg/kg) orally followed by intra-peritoneal (i.p.) administration of HMC (0.03–3 mg/kg) or vehicle. Plasmatic levels of urea, creatinine, oxidative stress, and cytokines were assessed. Regarding the kidneys, oxidative parameters, cytokine production, kidney swelling, urine NGAL, histopathology, and Nrf2 mRNA expression and downstream targets were evaluated. HMC dose-dependently targeted diclofenac systemic alterations by decreasing urea and creatinine levels, and lipid peroxidation, as well as IL-6, IFN-γ, and IL-33 production, and restored antioxidant properties in plasma samples. In kidney samples, HMC re-established antioxidant defenses, inhibited lipid peroxidation and pro-inflammatory cytokines and upregulated IL-10, reduced kidney swelling, urine NGAL, and histopathological alterations. Additionally, HMC induced mRNA expression of Nrf2 and its downstream effectors HO-1 and Nqo1, as well as reduced the levels of Keap1 protein detected in renal tissue. The present data demonstrate HMC is a potential compound for the treatment of acute renal damage caused by diclofenac, a routinely prescribed non-steroidal anti-inflammatory drug.

## 1. Introduction

The use of non-steroidal anti-inflammatory drugs (NSAIDs) is a common approach due to their analgesic, anti-inflammatory, and anti-pyretic effects [1]. Based on their recognized effectiveness for the treatment of inflammatory diseases and pain, their prescription is preferred in primary health care. However, NSAIDs can induce acute kidney injury (AKI) [2,3], a condition with potential health risks. Increased risk of AKI is observed in older individuals and in patients with chronic kidney disease [2]. Even in healthy patients, long term NSAID use may cause subclinical kidney dysfunction [4,5]. Diclofenac represents the most prescribed and used NSAID in low-, middle-, and high-income countries worldwide [6,7]. As a phenylacetic acid derivative, diclofenac is classified as a non-selective NSAID applied to treat pain, fever, and inflammation [1]. It is one of the main options for the treatment of acute and chronic pain, related mainly to the musculoskeletal system, including myalgia, lower back pain, osteoarthritis, rheumatoid arthritis, and ankylosing spondylitis [8,9,10]. Unfortunately, diclofenac has many adverse effects, such as gastrointestinal injury, hepatotoxicity, cardiovascular pathology, and AKI [1,3,9,11]. 

The main mechanism related to NSAID-induced AKI is prostaglandin inhibition, which in turn, has a fundamental role in the control of renin release, electrolytic dysfunction, and vasoconstriction [4,12]. At standard treatment doses, cyclooxygenase (COX)-2 selective and non-selective COX-2 NSAIDs induce a similar risk of AKI, depending on the study, and findings also suggest a higher risk upon the use of COX-2 NSAIDs with <5-fold selectivity compared to >5-fold selectivity [2]. Despite being considered as possessing equivalent inhibition of all COX enzymes, evidence also suggests diclofenac is more selective for COX-2 [7,13]. We previously demonstrated diclofenac does not alter COX-2 levels in renal tissue [9]. This particular aspect highlights the fact that diclofenac may induce AKI via a different primary pathological mechanism. It is postulated that a mechanism related to the induction of oxidative stress and/or reduction of antioxidant capacity may be determinant for diclofenac-induced nephrotoxicity [14,15]. In this sense, investigations have shown that through oxidative stress, increased cytokine release, and nuclear factor κB (NFκB) activation, diclofenac may induce AKI [1,9,16]. Therefore, the search for compounds that target these effector mechanisms, especially oxidative stress, is pertinent.

**Figure 1:**
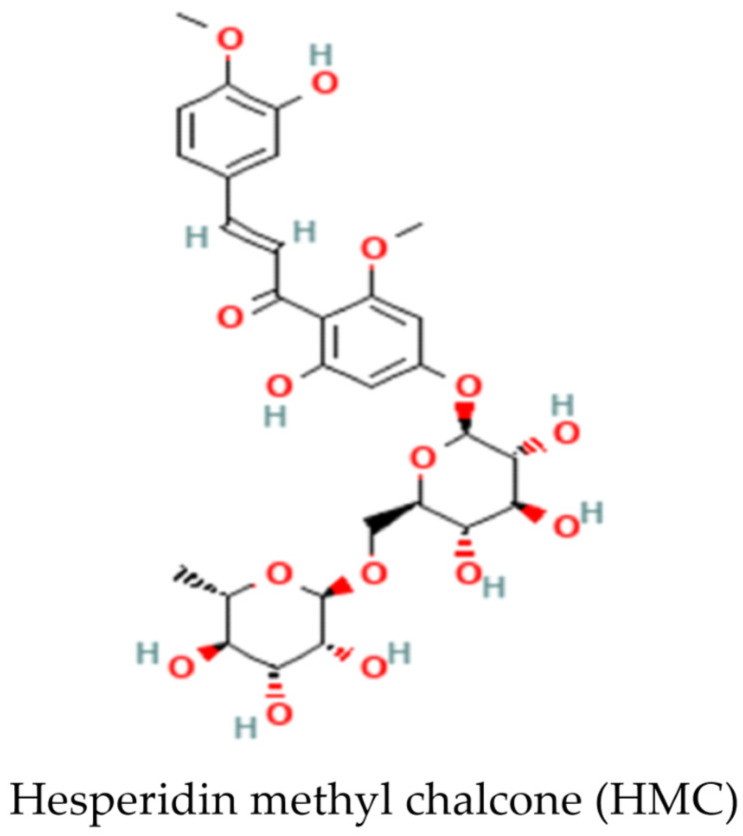
Chemical structure of HMC (compound CID: 6436550; https://pubchem.ncbi.nlm.nih.gov, accessed on 7 April 2022).

Hesperidin methyl chalcone (HMC; C_29_H_36_O_15_; Figure 1) is a product of methylation of the flavanone hesperidin (hesperidin-7-rhamnoglucoside), a flavonoid found in plants and foods, for instance, Rutaceae and citrus fruits, respectively [17,18]. Hesperidin presents poor water solubility, resulting in unsatisfactory absorption in the small intestine; however, its solubility is improved after a methylation reaction under alkaline conditions, which promotes hesperidin isomerization, and the generation of the HMC. Thus, the higher solubility of HMC confers enriched bioavailability, metabolic stability, and tissue absorption [19,20]. Methods applied to obtain HMC from hesperidin include methylation with dimethylsulfate [21]. Chromatography analysis indicates HMC is composed of both fully and partially methoxylated hesperidin, generated from methylation of the hydroxyl substituents on aglycon and linked sugars, characterizing this compound as a mixture of chalcones and flavanones species [21]. HMC peaks in the blood 1–2 h after oral administration and is excreted in both urine and feces within the first 24 h after administration [20]. In models of inflammation and pain, the biological properties of HMC include vasoprotective, antioxidant, anti-inflammatory, and analgesic effects [19,22,23,24,25]. HMC can both inhibit the major pro-inflammatory transcription factor nuclear factor κB (NFκB) [19,22,24] as well as induce nuclear factor erythroid 2-related factor 2 (Nrf2) signaling [23]. After the release of the Kelch-like ECH-associated protein 1 (Keap1)-Nrf2 complex in the cytoplasm, Nrf2 translocates to the nucleus where it activates nuclear antioxidant responsive elements (ARE) that regulate the dynamic expression of phase II genes, triggering the transcription of several detoxification enzymes and cytoprotective genes [26,27]. In humans, the efficacy of HMC in combination with other molecules for the treatment of vascular dysfunction, including hemorrhoid and chronic venous insufficiency, is supported by clinical trials [25,28,29,30]. Importantly, experimental and clinical data demonstrated that HMC is safe, even during long term use and high doses [31,32]. Thus, the eventual repurposing of HMC is feasible. Nevertheless, an investigation of its effects in kidney tissue stimulated with toxic doses of diclofenac, which mimics AKI induced by NSAIDs, has yet to be conducted. The present study aims to explore the beneficial therapeutic properties of HMC on experimental NSAID-induced AKI and the mechanisms underlying these effects.

## 2. Materials and Methods

### 2.1. Animals and Experimental Design

The study was carried out on Swiss mice (male, 30–35 g, aged 6–8 weeks) obtained through the State University of Londrina (Paraná State, Brazil). The conditions of the facility in which mice were maintained were as follows: ad libitum feed, twelve/twelve hours light/dark cycle, regular thermal comfort (21 °C), and circulation of air (15–30 cubic feet per minute/square feet). Animals were maintained in pathogen-free conditions. During the process of euthanasia for sample collection, animals were exposed to a lethal dose of 5% isoflurane, followed by cervical dislocation, and subsequent decapitation. The experimental protocol was carried out according to previous studies by our group [1,9]. Mice received a standard toxic dose of diclofenac (200 mg/kg, 100 μL, per oral), and 30 min later, were administered HMC (0.03, 0.3, and 3 mg/kg; 200 μL) or vehicle (saline; 200 μL) via the intra-peritoneal (i.p.) route. Blood, kidney, and urine samples were analyzed 24 h after NSAID (sodium diclofenac, SDCF) administration. Blood samples were collected after a dose-response experiment for the assessment of urea and creatinine levels to determine the best dose of HMC, and a dose of 3 mg/kg of HMC was chosen for all subsequent experiments in the study. After this initial analysis, blood samples were collected for a new round of experiments that included evaluations of oxidative stress (antioxidant capacity parameters and lipid peroxidation) and cytokine production (both described in detail below). Renal tissue and urine samples were collected to evaluate the following parameters: oxidative stress (antioxidant capacity parameters and lipid peroxidation), cytokine production, swelling, histopathological changes, and mRNA expression of Nrf2 and its downstream effectors, as well as the concentration of a well-known urinary marker of AKI (NGAL). The experiments using kidney samples were conducted on the entire organ (one kidney per analysis).

### 2.2. Compounds Used in the Study

SDCF (Neutaren^®^) was purchased from Novartis (São Paulo, SP, Brazil); HMC was acquired from Santa Cruz Biotechnology (Santa Cruz, CA, USA); and saline was acquired from Gaspar Viana S/A (Fortaleza, CE, Brazil). The dilution of SDCF and HMC using saline was performed immediately before administration via the oral (p.o.) and i.p. routes, respectively. 

### 2.3. Evaluation of Renal Function Markers

The evaluation of plasma concentrations of urea and creatinine were performed in blood samples after p.o. administration of SDCF (24 h). Samples were collected into heparinized tubes with posterior centrifugation (200× *g*, 10 min, 4 °C), and subsequently processed for determination of renal function markers using commercial kits (Labtest Diagnóstico S.A., Lagoa Santa, MG, Brazil). The data are shown as milligram per deciliter (mg/dL) of plasma.

### 2.4. Ferric-Reducing Ability Potential (FRAP), 2,2′-Azino-bis(3-ethylbenzothiazoline-6-sulfonic Acid) (ABTS^•+^) Radical Cation, and Reduced Glutathione (GSH) Assays

FRAP, ABTS and GSH assays were performed to evaluate antioxidant capacity during the protocols in the present model [1,9]. Kidney and blood samples (EDTA microtubes) were collected 24 h after SDCF administration and homogenized with 500 μL of 1.15% KCl, subsequently centrifuged (10 min × 200× *g* × 4 °C), and the ability of the sample to resist oxidative damage was determined by measuring ferric-reducing ability with the FRAP assay and free radical scavenging ability with the ABTS assay. FRAP determination used 50 μL of supernatant, together with 150 μL of deionized water and 1.5 mL of freshly prepared FRAP reagent. The reaction mixture was incubated at 37 °C for 30 min, and subsequently, the absorbance was measured at 595 nm. The ABTS test was conducted by using ABTS solution diluted with phosphate-buffered saline at pH 7.4 to an absorbance of 0.80 at 730 nm. After this step, 1.0 mL of diluted ABTS solution was mixed with 20 μL of supernatant (as prepared for the FRAP assay). After 6 min, the absorbance was measured at 730 nm. The results were equated against a Trolox standard curve (1.5–30 μmol/L, final concentrations). The results are expressed as nanomoles (nmol) of Trolox equivalents per milliliter (mL) or milligram (mg) of tissue for plasma and kidney, respectively, for both analyses. For the GSH assay, kidney samples were harvested 24 h after SDCF administration. Samples were homogenized in 0.02 M ethylenediamine tetraacetic acid (EDTA) reagent and treated with 2 mL of water plus 0.5 mL of 50% TCA (trichloroacetic acid). Next, homogenates underwent centrifugation (1500× *g*, 15 min, 4 °C) and the resultant supernatants were carefully removed for subsequent addition to 2 mL of Tris 0.4 M (pH 8.9) plus 50 mL of dithionitrobenzoic acid (DTNB) solution. After 5 min, spectrophotometric readings were carried out at 412 nm. Data are expressed as nmol of GSH per mg of tissue. For the three analyses, a Multiskan GO Microplate Spectrophotometer (Thermo Scientific, Vantaa, Finland) was used. 

### 2.5. Assessment of Thiobarbituric Acid-Reactive Substances (TBARS)

Lipid peroxidation in kidney and blood samples (EDTA microtubes) was assessed 24 h after the administration of SDCF via TBARS determination using an adapted methodology described previously [9]. In brief, TCA (10%) was included in the tissue homogenate or plasma samples to precipitate the proteins. Subsequently, samples underwent centrifugation (1000× *g*, 3 min, 4 °C) and the supernatant was removed for the next step. The supernatants were then mixed with thiobarbituric acid (TBA; 0.67%), incubated for 15 min in a boiling water bath (100 °C), then transferred to an ice bath. Malondialdehyde (MDA) was then quantitated as an indicator of lipid peroxidation in kidney and plasma samples by measuring the absorbance by spectrophotometry (572–535 nm). Data are presented as TBARS (nmol of MDA per mL) for plasmatic samples, and as TBARS (nmol of MDA per mg of tissue) for renal samples.

### 2.6. Evaluation of Cytokines and Neutrophil Gelatinase-Associated Lipocalin (NGAL) Production

The following cytokines were assessed in blood and kidney samples 24 h after the administration of SDCF: interleukin (IL)-1β, IL-6, interferon (IFN)-γ, IL-33, and IL-10. Considering plasmatic assay, after collection (EDTA microtubes), samples were centrifuged (800× *g*, 10 min, 4 °C), and the generated supernatants were used to assess the levels of cytokines. Kidney samples were homogenized in 500 μL of saline. Cytokine levels in both tissues were then measured using enzyme-linked immunosorbent assays (ELISA) according to the manufacturer’s instructions (eBioscience, San Diego, CA, USA) and analyzed spectrophotometrically. Data are expressed as mg/dL for plasma samples and as picograms (pg) per 100 mg of tissue for kidney samples [1,9]. NGAL urine level was also evaluated by ELISA 24 h after the administration of SDCF. Urine samples were collected into EDTA microtubes after applying moderate compression of the pelvic region of mice. Samples were then transferred into anti-mouse NGAL pre-coated plates and processed according to the manufacturer’s instructions (Cloud-Clone Corp., Katy, TX, USA). The levels of NGAL were analyzed by spectrophotometry at 450 nm, and the data are presented as nanogram (ng) per mL of urine [1].

### 2.7. Histopathological and Swelling Evaluations

For histopathological analysis, kidneys were collected 24 h after the administration of SDCF. Kidneys initially underwent a fixation process using 4% paraformaldehyde (PFA) in phosphate-buffered saline (PBS). Subsequently, the kidneys were dehydrated in a graded series of ethanol solutions and finally processed for paraffin embedding. The process of sectioning the cortical potions of the organs was carried out using a cryostat (CM1520, Leica Biosystem, Richmond, IL, USA) with a thickness of 5 μm. After this step, for the sections underwent hematoxylin and eosin (H&E) and periodic acid–Schiff (PAS) staining. Stained sections from the control group, model group (SDCF) treated with vehicle, and model group treated with HMC were analyzed in a blinded manner through the use of light microscopy at 40× magnification. A semi-quantitative assessment of kidney damage was carried out in 10 high-power fields randomly selected as described previously with modifications [1,9,33] with scoring for each animal. Summed histopathological scores of different experimental groups were determined by the morphological analysis of the following parameters: (1) glomerular pathology; (2) impairment of the cortical brush border; and (3) the presence of vacuoles in tubular cells. A four-point scale was used to describe the level of pathological change: 0, normal; 1, mild; 2, moderate; 3, severe. The score for each parameter were combined into a total histopathological score (9 maximum). Kidney swelling was also evaluated 24 h after SDCF administration by using the organ wet weight as an indicator. After collection, the kidneys were weighed on a precision balance and the results presented as mg of renal tissue per gram (g) bodyweight.

### 2.8. Reverse Transcription and Quantitative Polymerase Chain Reaction (RT-qPCR) Assay 

RT-qPCR was performed as previously described [34]. Kidneys were collected 24 h after the administration of SDCF, homogenized in TRIzol™ Reagent (Thermo Fisher Scientific, Waltham, MA, USA), and total RNA was isolated according to the manufacturer’s guidelines. The purity of total RNA was measured spectrophotometrically (Multiskan GO Microplate Spectrophotometer, Thermo Scientific, Vantaa, Finland), and the wavelength absorption ratio (260/280) was between 1.8 and 2.0 for all preparations. Reverse transcription of total RNA to cDNA and qPCR were performed using the GoTaq^®^ 2-Step RT-qPCR System (Promega, Madison, WI, EUA) and target primers with the Step One Plus TM Real-Time PCR System (Applied Biosystems^®^, Waltham, MA, USA). The relative gene expression was measured using the comparative 2^−(ΔΔCq)^ method. The primers used in this study were as follows: Nrf2—forward, 5′-TCACACGAGATGAGCTTAGGGCAA-3′; reverse, 5′-TACAGTTCTGGGCGGCGACTTTAT-3′ (gene accession number 18024); heme-oxygenase-1 (Ho-1)—forward, 5′-CCCAAAACTGGCCTGTAAAA-3′; reverse, 5′-CGTGGTCAGTCAACATGGAT-3′ (gene accession number 15368); NAD(P)H dehydrogenase (quinone 1) (Nqo1)—forward, 5′-TGGCCGAACACAAGAAGCTG-3′; reverse, 5′-GCTACGAGCACTCTCTCAAACC-3′ (gene accession number 18104). The expression of β-actin (forward, 5′-AGCTGCGTTTTACACCCT TT-3′; reverse, 5′-AAGCCATGCCAATGTTGTCT-3′ (gene accession number 11461) mRNA was used as a control for tissue integrity in all samples.

### 2.9. Immunofluorescence Assay in Confocal Microscopy

Twenty-four hours after the administration of SDCF, animals underwent a perfusion process using 4% PFA in PBS injected via the ascending aorta artery. Next, the kidney was carefully removed and immersed in 4% PFA and remained in this solution for the next 24 h. After this period, samples were placed in 30% saccharose and incubated for 3 days. Once embedded (Tissue-Tek^®^ reagent, Torrance, CA, USA), the kidneys were sectioned to a thickness of 10 µm using cryostat equipment (CM1520, Leica Biosystems, Wetzlar, Germany). Four samples per animal per slide and five animals per group were analyzed. Antigenic recovery was performed (exposure to 90 °C followed by immediate cooling until 30 °C) then the sections passed through a blocking stage (200 µL/slide; 0.5% tween 20 and 5% bovine serum albumin in PBS) for 2 h, followed by overnight incubation at 4 °C with the primary antibody (keap1, D6B12, rabbit IgG mAb, #8047, 1:100 dilution, Cell Signaling Technology, Danvers, MA, EUA). A solution containing the secondary antibody (anti-rabbit IgG Fab2 Alexa Fluor^®^ 647, #4414S, 1:1000 dilution, Cell Signaling Technology, Danvers, MA, EUA) was applied to the slides the next day for 1 h. Treatment with secondary antibody alone was used to test for non-specific staining. For assembly of the slides, DAPI melting media reagent (ProLong^TM^ Gold Antifade Mountant, #P36931, Thermo Fisher Scientific, Waltham, MA, USA) was used. Immunofluorescence analysis of aleatory fields using a confocal microscope (TSC SP8 Leica microsystem, Wetzlar, Germany) were performed on different portions of the cortical region of kidneys with a magnification of 40×. Representative images from each group are presented with a 50 µm scale. Keap1 fluorescence intensity were analyzed by a blinded experimenter and measured using confocal microscope software to provide quantitative data for the experiment.

### 2.10. Statistical Methodology

Statistical methods were applied to 6 animals per group (5 animals for immunofluorescence) in individual experiments. For histopathological evaluations, the final score considered 12 animals per group in individual experiments. The results are representative of two independent experiments. One-way analysis of variance with Tukey’s post hoc test was used for the determination of statistical interpretations. Additionally, the non-parametric Kruskal–Wallis test with Dunn’s post hoc tests was applied to the analysis of categorical variables. The analyses were carried out with GraphPad Prism 7.00 (GraphPad software Inc., La Jolla, CA, USA) software. All data are presented as the mean ± standard deviation (SD). Results with values of *p* < 0.05 were considered statistically significant.

## 3. Results

### 3.1. HMC Reduces SDCF-Triggered Renal Dysfunction: Urea and Creatinine Levels, and Oxidative Stress in Plasma

**Figure 2:**
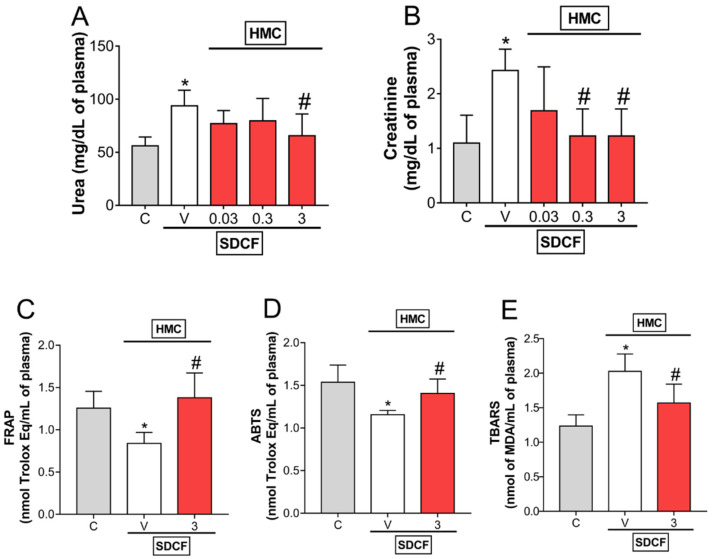
HMC inhibits SDCF-induced increase of plasmatic levels of urea, creatinine, and oxidative stress. Blood samples were collected 24 h after the administration of SDCF for the evaluation of urea (**A**), creatinine (**B**), FRAP (**C**), ABTS (**D**), and TBARS (**E**) levels. Results are expressed as mean ± SD, *n* = 6 mice per group per experiment, and are representative of two independent experiments. * *p* < 0.05 vs. control (C) group; # *p* < 0.05 vs. vehicle (V) treated group; one ANOVA followed by Tukey’s post hoc test.

Our first approach was designed to determine the most effective dose of HMC to inhibit SDCF-induced renal dysfunction. SDCF was administrated to the mice orally, and after 30 min, they received i.p. treatment with HMC (0.03, 0.3, and 3 mg/kg). The plasmatic levels of urea and creatinine (Figure 2A,B, respectively) were determined 24 h later. HMC treatment inhibited the elevation of renal dysfunction markers induced by SDCF in a dose-dependent manner. For urea, it was observed that only a dose of 3 mg/kg inhibited the increase induced by SDCF. For creatinine, intermediate and high doses of HMC (0.3, and 3 mg/kg, respectively) inhibited the effect of SDCF. Since 3 mg/kg was the only dose able to inhibit both markers of impaired renal function, this dose was selected for the following experiments. Thereafter, we investigate the antioxidant properties of HMC upon SDFC-induced oxidative stress (Figure 2C–E). HMC treatment restored the impaired plasmatic antioxidant status induced by SDFC, seen as increased FRAP and ABTS levels compared to the vehicle control, and inhibited lipid peroxidation levels, seen as a reduced concentration of TBARS. These results indicate HMC protects renal tissue from the toxic effects of SDFC. Further, HMC reduces systemic oxidative parameters in AKI mice, which reflects its potential antioxidant actions in response to increased free radical activity.

### 3.2. HMC Reduces IL-6, IFN-γ, and IL-33, but Does Not Modify IL-1β and IL-10 Levels in Plasma

**Figure 3:**
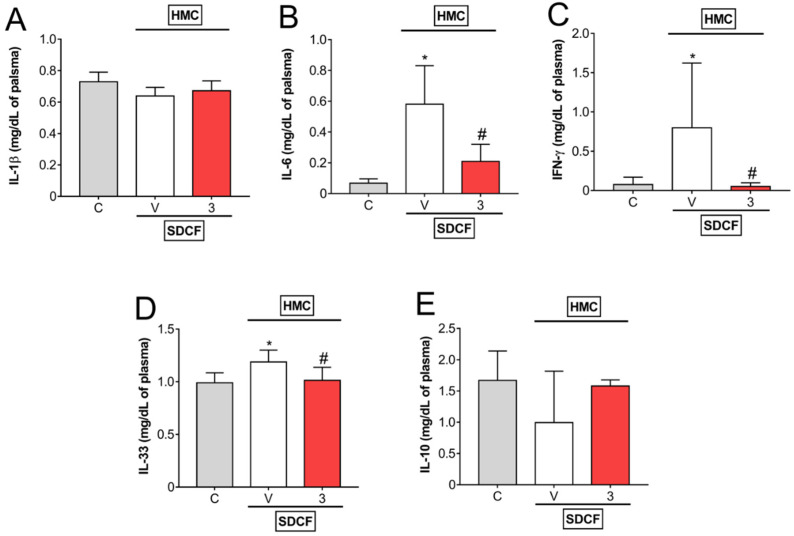
HMC inhibits SDCF-induced IL-6, IFN-γ, and IL-33, but does not change IL-1β and IL-10 plasmatic levels. Blood was collected 24 h after the administration of SDCF for the evaluation of IL-1β (**A**), IL-6 (**B**), IFN-γ (**C**), IL-33 (**D**), and IL-10 (**E**) levels. Results are expressed as mean ± SD, *n* = 6 mice per group per experiment, and are representative of two independent experiments. * *p* < 0.05 vs. control (C) group; # *p* < 0.05 vs. vehicle (V) treated group; one ANOVA followed by Tukey’s post hoc test.

The next investigation aimed to evaluate the levels of pro- and anti-inflammatory cytokines in plasma. For this approach, SDCF was administrated to the mice orally, and after 30 min, they received i.p. treatment with HMC (3 mg/kg) for the evaluation of plasmatic levels of IL-1β, IL-6, IFN-γ, IL-33, and IL-10 (Figure 3A–E). SDCF did not interfere with IL-1β and IL-10, however, it induced a significant increase in IL-6, IFN-γ, and IL-33 levels in plasma. HMC treatment did not affect IL-1β and IL-10, but inhibited SDCF-induced IL-6, IFN-γ, and IL-33 (Figure 3A–E). These data suggest HMC may modulate some pro-inflammatory cytokines systemically in AKI mice.

### 3.3. HMC Reduces Oxidative Stress in Renal Tissue 

**Figure 4:**
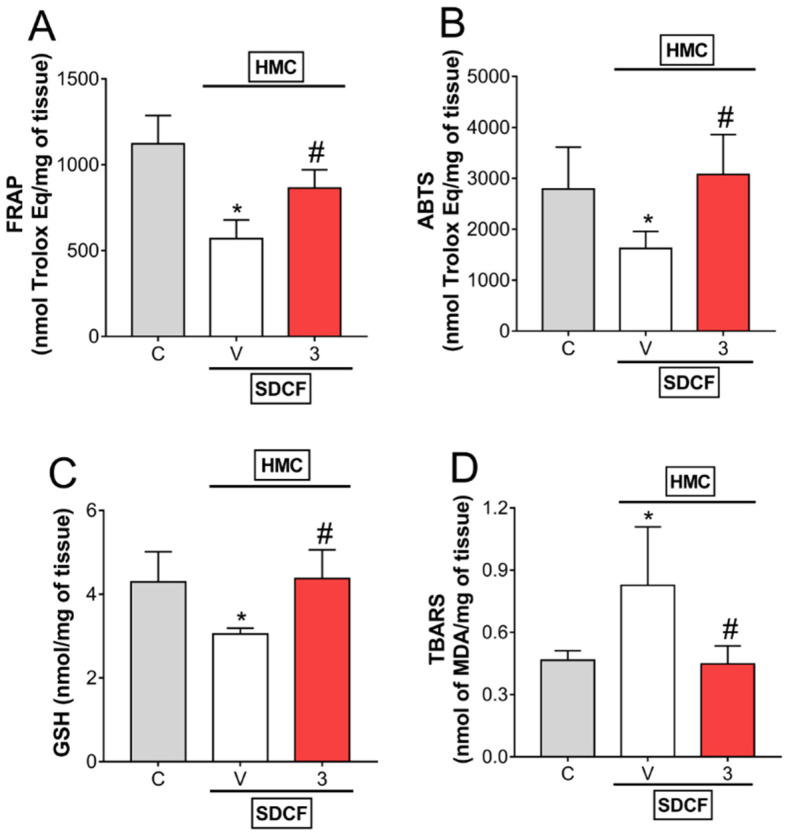
HMC inhibits SDCF-induced oxidative stress in renal tissue. Kidney was collected 24 h after the administration of SDCF for the evaluation of FRAP (**A**), ABTS (**B**), GSH (**C**), and TBARS (**D**) levels. Results are expressed as mean ± SD, *n* = 6 mice per group per experiment, and are representative of two independent experiments. * *p* < 0.05 vs. control (C) group; # *p* < 0.05 vs. vehicle (V) treated group; one ANOVA followed by Tukey’s post hoc test.

Antioxidant parameters and lipid peroxidation levels were measured in the kidneys, the target organ for oxidative stress induced by SDCF, to investigate the effects of HMC. SDCF was administrated to the mice orally, and after 30 min, they received i.p. treatment with HMC (3 mg/kg) for the evaluation of FRAP, ABTS, GSH, and TBARS levels (Figure 4A–D). The toxic dose of SDCF impaired antioxidant defenses, observed as reduced FRAP, ABTS, and GSH levels, and increased lipid peroxidation, observed as increased TBARS levels in renal tissue. HMC treatment re-established all antioxidant parameters, and even inhibited lipid peroxidation in kidneys (Figure 4A–C). These data demonstrate HMC can effectively counteract the oxidative stress induced by SDCF in renal tissue.

### 3.4. HMC Reduces IL-1β, IL-6, IFN-γ, and IL-33, as well as Increases IL-10 Levels in Renal Tissue

**Figure 5:**
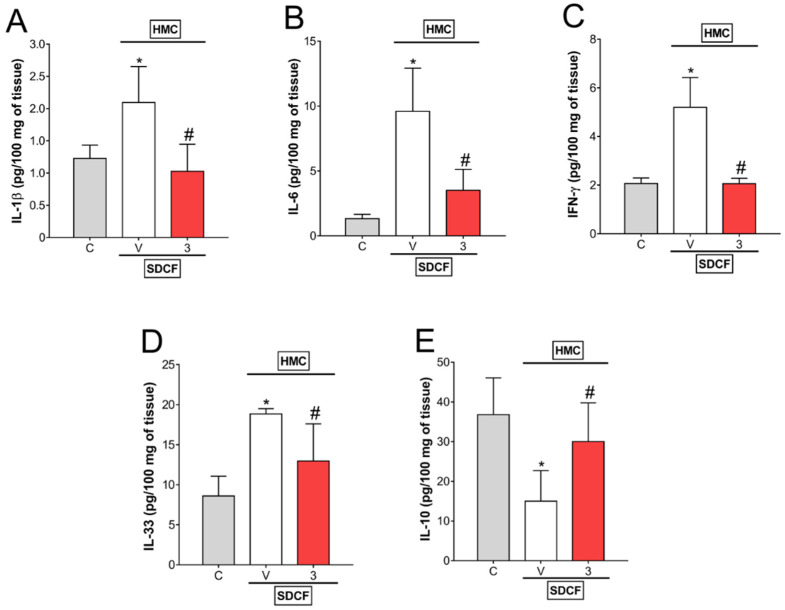
HMC inhibits SDCF-induced IL-1β, IL-6, IFN-γ, and IL-33, and reverses SDCF-induced depletion of IL-10 levels in renal tissue. Kidney was collected 24 h after the administration of SDCF for the evaluation of IL-1β (**A**), IL-6 (**B**), IFN-γ (**C**), IL-33 (**D**), and IL-10 (**E**) levels. Results are expressed as mean ± SD, *n* = 6 mice per group per experiment, and are representative of two independent experiments. * *p* < 0.05 vs. control (C) group; # *p* < 0.05 vs. vehicle (V) treated group; one ANOVA followed by Tukey’s post hoc test.

After determining the systemic modulation of cytokines by HMC in SDCF-induced AKI, our next objective was to evaluate the modulation of cytokines by HMC in renal tissue. Therefore, SDFC was administrated to the mice orally, and after 30 min, they received i.p. treatment with HMC (3 mg/kg) and IL-1β, IL-6, IFN-γ, IL-33, and IL-10 levels were determined in renal tissue (Figure 5A–E). SDCF increased the production of pro-inflammatory cytokines IL-1β, IL-6, IFN-γ, and IL-33, and reduced the production of the anti-inflammatory cytokine IL-10. Treatment with HMC efficiently inhibited the increased levels of IL-1β, IL-6, IFN-γ, and IL-33 induced by SDCF, and restored the levels of IL-10 significantly (Figure 5A–D). These results indicate that in addition to inhibiting oxidative stress, HMC acts by inhibiting pro-inflammatory and inducing anti-inflammatory cytokines to combat the toxic effects of SDCF in the kidney. 

### 3.5. HMC Reduces SDCF-Induced Renal Histopathology, Swelling and Tubular Cells Cytotoxicity

**Figure 6:**
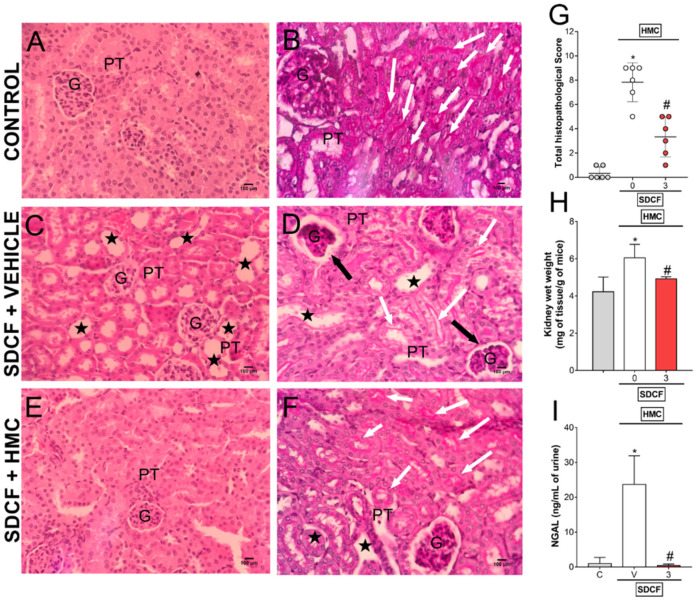
HMC inhibits SDCF-induced renal histopathology, swelling and tubular cells cytotoxicity. Kidney samples were collected 24 h after the administration of SDCF for the evaluation of histopathology with H&E (**A**,**C**,**E**), and PAS (**B**,**D**,**F**) staining, total histopathological score (**G**), swelling (**H**), and NGAL urinary levels (**I**). Original magnification 40×; 100 µm scale. Stars show tubular dilatation; black arrows show glomeruli/Bowman’s capsule lesions; and white arrows show brush border differences in varied experimental groups. Data are shown as mean ± SD, *n* = 12 and *n* = 6 mice per group per experiment for histopathological analysis and swelling/NGAL, respectively, and are representative of two independent experiments. * *p* < 0.05 vs. control (C) group; # *p* < 0.05 vs. SDCF + vehicle (V) treated group; Kruskal-Wallis followed by Dunn’s post hoc test (**G**) and one ANOVA followed by Tukey’s post hoc test (**H**,**I**). G, glomerulus; PT, proximal tubule.

Our next goal was to investigate the protective effects of HMC upon tissue inflammatory pathology induced by SDCF. For this approach, SDCF was administrated to the mice orally, and after 30 min, they received i.p. treatment with HMC (3 mg/kg) for the evaluation of renal histopathology and swelling, and NGAL urinary levels (Figure 6). SDCF altered the regular morphology of the cortical layer of renal tissue, observed as tubular cell dilatation together with flattening of the renal epithelium and disruption of the brush borders in the proximal convoluted tubes, as well as deformation in glomeruli shape and Bowman’s capsule injury (Figure 6C,D, respectively), which were not observed in control mice (Figure 6A,B, respectively). HMC treatment reduced this altered morphology, conferring protection on the kidney (Figure 6E,F). Besides reducing the histopathology in renal tissue (Figure 6G), HMC treatment also inhibited kidney swelling and reduced NGAL levels in urine (Figure 6H,I, respectively), which denotes a reduction in organ inflammation and tubular cells damage. Altogether, these data show HMC can act as a powerful therapeutic compound for SDCF-induced AKI-related tissue pathology. 

### 3.6. HMC induces Nrf2 Signaling to Reduce SDCF-Induced AKI

**Figure 7:**
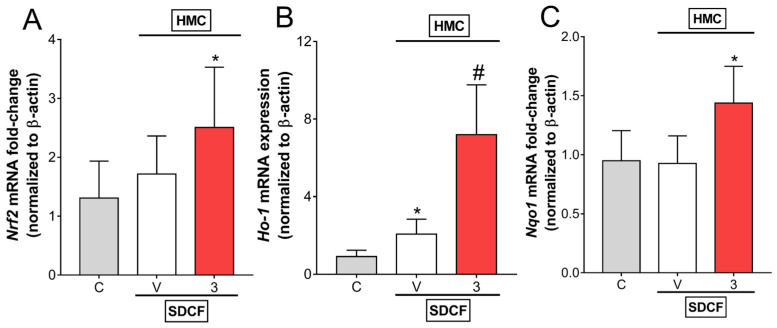
HMC induces Nrf2 signaling in renal tissue. Kidney samples were collected 24 h after the administration of SDCF for the evaluation of Nrf2 (**A**), Ho-1 (**B**), and Nqo1 (**C**) mRNA expression. Results are expressed as mean ± SD, *n* = 6 mice per group per experiment, and are representative of two independent experiments. * *p* < 0.05 vs. control (**C**) group; # *p* < 0.05 vs. vehicle (V) treated group; one ANOVA followed by Tukey’s post hoc test.

Considering the importance of oxidative stress to SDCF-triggered AKI and HMC activity, we investigated whether the HMC protective mechanism involves the activation of the major antioxidant pathway, Nrf2/ARE. Therefore, SDCF was administrated to the mice orally, and after 30 min, they received i.p. treatment with HMC (3 mg/kg) for the evaluation of Nrf2, HO-1, and Nqo1 mRNA expression (Figure 7). Nrf2 and Nqo1 mRNA expression were not altered by SDCF administration (Figure 7A,C, respectively), however Ho-1 expression was increased by SDCF (Figure 7B). Treatment with HMC significantly increased Nrf2 and Nqo1 mRNA expression compared to control mice, and more robustly, increased Ho-1 mRNA expression in comparison to both control and SDCF administered mice (Figure 7A–C). These results demonstrate the induction of the Nrf2 pathway by HMC, and consequently, its downstream signaling effectors, contributing to the mechanism that reduces SDCF-induced AKI. 

### 3.7. HMC Reduces Keap1 in the Kidney

**Figure 8:**
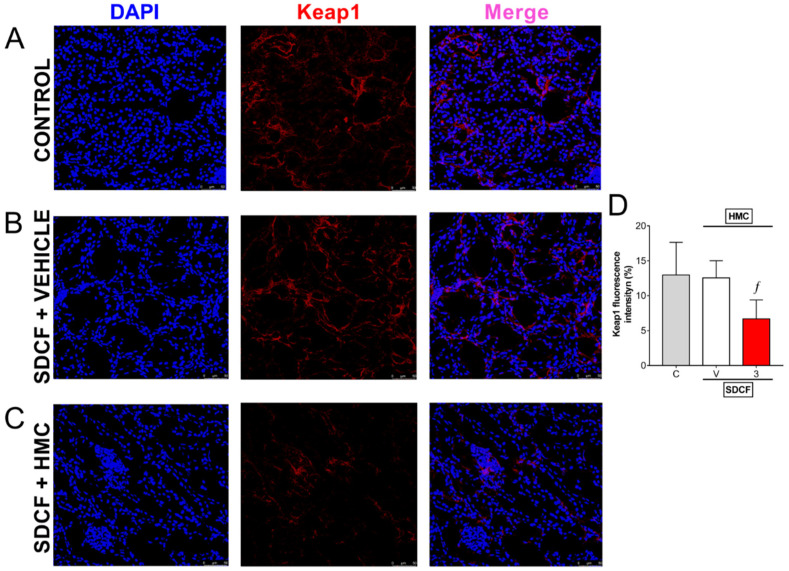
HMC reduces Keap1 protein expression in renal tissue. Kidney samples were collected 24 h after the administration of SDCF for the evaluation of immunofluorescence detection of Keap1 (**A**–**D**). Original magnification 40×; 50 µm scale. DAPI was used for nuclear detection in samples. Data are showed as mean ± SD, *n* = 5 mice per group per experiment, and are representative of two independent experiments. ^*f*^ *p* < 0.05 vs. control (C) and SDFC + vehicle treated (V) groups; one ANOVA followed by Tukey’s post hoc test.

The results in Figure 7 indicate that the Nrf2 system is stimulated by HMC treatment. Keap1 is a negative regulator of Nrf2 present in the cytoplasm. Keap1 favors cullin-based E3 ubiquitin ligase-mediated ubiquitination of Nrf2 [26]. Control and SDCF + vehicle groups presented similar renal staining for Keap1 indicating that Nrf2 is under control (Figure 8A,B,D). HMC treatment reduced Keap1 fluorescence detection (Figure 8C,D), and Nrf2 would be able to translocate to the nucleus and activate ARE-dependent gene expression in these mice. Thus, these data line up with the previous results indicating HMC reduces oxidative stress and enhances endogenous antioxidant defenses as well as stimulating the Nrf2 pathway and its downstream targets.

## 4. Discussion

Although considered an effective pharmacological tool for the treatment of fever, acute and chronic pain, and inflammatory diseases, the clinical applicability of diclofenac is frequently hampered by adverse effects related to its use [1,3,9,11]. Kidneys represent the master human organ related to diclofenac excretion [35]. For this reason, renal tissue is frequently exposed to diclofenac and its metabolites, such as diclofenac acyl glucuronide (diclofenac beta-D-glucosiduronic acid; C_20_H_19_Cl_2_NO_8_), and thus, is especially vulnerable to their toxic effects [36]. The nephrotoxic effects of diclofenac are dose-dependent, increasing concomitantly with higher doses [37]. Moreover, the interaction of diclofenac with other drugs, including the nucleotide analogue inhibitor of reverse transcriptase, tenofovir disoproxil, substantially boosts the risk of acute kidney injury [38]. Evidence also indicates that long-term use together with the analgesic drug paracetamol (acetaminophen) leads to drug-induced chronic kidney disease [39]. All these data highlight the need for new alternative therapies to treat AKI.

The present study demonstrates for the first time the nephroprotective effects of the flavonoid HMC in diclofenac-induced AKI in mice. HMC reversed the dysfunctional pathological aspects of AKI since we observed an improvement in the levels of the renal function markers urea and creatinine. The magnitude of inflammation in acute kidney injury may vary according to some aspects, including age and weight of animals [40,41]. Kidney inflammation caused by SDCF was also counteracted by HMC, reducing kidney swelling and modulating systemic and renal cytokine production. Mechanistically, we showed HMC presents a remarkable antioxidant effect in blood and kidney (restoration of antioxidant capacity and reduction of lipid peroxidation), with this effect being attributed to the structural antioxidant activity of HMC [23] and activation of the Nrf2 pathway. The outcomes observed in the present model indicated diminished damage to kidney tissue after SDCF administration (attenuation of renal histopathological score and NGAL urinary levels in HMC treated mice). The HMC dose needed to achieve these effects in the present intoxication model was 3 mg/kg. In other models studied by our group, including those of inflammation and pain, treatment effects were obtained with higher doses [19,22,24,35,42]. The difference among these models is a major point for the difference in HMC action. Models in which an inflammatory stimulation activated tissue resident and recruited immune cells through receptors to cause inflammation and pain characterizes our previous studies. The current SDCF-induced AKI is a different condition because it is not related to the primary mechanism of action for SDCF, that is, the inhibition of COX isoforms. SDCF does not induce oxidative stress at therapeutic doses. However, there is overuse and intentional intoxication on some occasions. Thus, it is essential to note that in previous studies we adopted models based on pharmacology principles in which the recruitment and activation of leukocytes was higher, and consequently, oxidative stress was also higher. It is hypothesized that SDCF and its metabolites cause kidney damage via interaction with renal organic anion transporters (OATs) [36], a different mechanism to that induced by inflammatory stimuli. Furthermore, our previous studies were of arthritis, skin inflammation and colitis, thus, the targets tissues involved and the physiopathological mechanisms in each model were different, as were the stimuli. Additionally, the routes used for the administration of HMC varied between these studies (oral and i.p.), which modifies the pharmacokinetics of the drug, as well as its bioavailability. In addition to all these differences, it is important to highlight that HMC is excreted is the urine [20], likely allowing more abundant accumulation of the compound in the kidney. Thus, we speculate that it may reach higher concentrations in the kidney than in other organs, such as joints and skin, which were used in previous studies. Therefore, these variables (different stimuli, physiopathological mechanisms, affected tissues, disease duration, route of excretion, and routes of HMC administration of) may explain the different dosages needed for a treatment effect among these studies.

Acute renal failure is clinically observed as a rapid elevation in serum creatinine and urea concentrations above the limits considered normal. The main rationale characterizing the use of urea and creatinine levels as markers of AKI concerns glomerular filtration rate (GFR) status, a fundamental aspect for clinical diagnosis of AKI. As GFR declines, the excretion of urea and creatinine in urine decreases and blood concentrations increase [43]. We observed a clear glomerular architectural change after SDCF administration together with increased urea and creatinine levels in the blood, indicating glomerulus injury and reduced GFR, respectively. These changes were inhibited in mice that received HMC treatment, indicating this flavonoid targets SDCF toxicity to prevent functional deficits in renal tissue. Importantly, although frequently used, urea and creatinine serum levels may not be as sensitive for identifying AKI [1,43]. Thus, we are also concerned with evaluating the most reliable markers for kidney damage. Preclinical studies were very important for the discovery of more specific markers of kidney injury [44]. NGAL protein is considered a sensitive and predictive early molecule of AKI [45], and its urinary increase reflects damage, especially to the glomeruli and proximal tubules [46,47]. In a previous study by our group, we demonstrated for the first time that NGAL is also an important marker of SDCF-induced AKI [1]. In this sense, we evaluated the effects of HMC on SDCF-induced increased NGAL urinary levels. HMC treatment efficiently mitigated the rise in NGAL levels, which is consistent with the improvement in renal function (reduced urea and creatinine levels) and histopathological score (reduced glomerular and proximal tubular cells damage) observed in HMC-treated mice. 

After observing that HMC leads to reduced SDCF toxicity in renal tissue, the mechanisms by which HMC confers such protection were investigated. As mentioned earlier, HMC is known for its anti-inflammatory and antioxidant effects. SDCF induces the activation of NFκB in the kidney [1,9,48] and leads to an increase in the production of inflammatory mediators, including cytokines [1,9]. In AKI, cytokines may be released by recruited and/or resident leukocytes as well as by renal tubular cells, promoting kidney inflammation (as observed by kidney swelling in the present study). Cytokines are also released into the blood, thus reflecting potential urine and blood biomarkers of AKI [1,9,49,50]. Their systemic release during AKI may even promote damage to distant organs, raising the importance of inhibiting cytokine production to avoid both kidney and distant organ injury [49]. We observed that HMC inhibited pro-inflammatory cytokine production and stimulated an anti-inflammatory cytokine after SDCF administration. In plasma samples, HMC inhibited IL-6, IFN-γ, and IL-33 levels, whereas it did not affect IL-1β and IL-10 levels since they were not altered in SDCF AKI. In the kidney, the inhibition detected after HMC treatment included IL-6, IFN-γ, IL-33, and IL-1β. The profile for IL-10 levels in plasma and renal tissue differed between the experimental groups. In the plasma, there was only a tendency for a reduction in IL-10 in the SDCF vehicle-treated group, and for an increase in the HMC-treated group, which contrasts with the significant changes observed in renal tissue. This apparent incongruence in the data may represent differences in the dynamics of cytokine production and release after SDCF stimulus. Regardless, HMC inhibited all evaluated pro-inflammatory cytokines altered by SDCF, and at the same time, it induced IL-10 in the kidney. In renal tissue, an increase in IL-10 production is interesting considering that besides being a fundamental cytokine for controlling excessive inflammation through inhibition of pro-inflammatory cytokines, IL-10 may positively regulate HO-1 [51]. In turn, HO-1 promotes adaptive antioxidant cellular response to reduce or prevent damage resulting from oxidative stress. In SDCF-induced AKI, we demonstrated that in addition to inducing IL-10 production, HMC also activated another decisive signaling cascade that mediates HO-1 production, the Nrf2/ARE antioxidant pathway, which is discussed below.

Mice that were treated with HMC presented increased antioxidant capacity in both plasma and renal samples, as indicated by FRAP and ABTS tests. Reduced levels of lipid peroxidation were also detected in both tissues in animals treated with HMC. Moreover, HMC induced increased production of the non-enzymatic antioxidant GSH in renal tissue. These data are extremely important, since oxidative stress accounts for the impairment in GFR [52,53]. In fact, in a reactive oxygen species-dependent manner, cytokines such as IL-1β and IL-6 may promote dysfunction of glomerular permeability to impair the GFR rate [53]. These latter data highlight the intimate link between cytokines and oxidative stress in renal damage caused by SDCF. The present results corroborate previous studies which demonstrated potential antioxidant effects of HMC in other models involving different pathological mechanisms [19,22,23,24,54]. For instance, in ultraviolet B (UVB)-irradiated mouse skin, HMC restored impaired GSH production and inhibited the expression of gp91^phox^ subunit of nicotinamide adenine dinucleotide phosphate (NADPH) oxidase that generates superoxide anions [23,54]. Increased production of cellular superoxide anion is a contributing mechanism to the perpetuation of an oxidant cascade that ultimately leads to lipid peroxidation. The restoration of GSH levels and inhibition of oxidative stress by HMC were also demonstrated in models of zymosan-induced arthritis [22] and experimental ulcerative colitis [42]. In the present experimental model, HMC efficiently reduced lipid peroxidation in blood and renal tissues. HMC has the structural ability to act as an antioxidant [23]; however, the activation of the Nrf2/ARE signaling pathway might also account for the effects of HMC. HMC can induce Nrf2 signaling in inflamed skin [23], and here, we demonstrate this modulation can also occur in the kidney after suffering the toxic effects of SDCF. ARE-dependent gene expression drives the canonical expression of HO-1, NQO1, glutamatecysteine ligase (GCL), glutathione S-transferases (GSTs), catalase (CAT), superoxide dismutase (SOD), and thioredoxin, among others [26], which mediate powerful antioxidant effects. Through GCL induction, Nrf2 can upregulate GSH levels [55]. Therefore, the upregulation of GSH observed here after HMC treatment is consistent with an effect on Nrf2 activity. In addition to increasing GSH levels in renal tissue, HMC effectively enhanced mRNA expression for *Nrf2* and its downstream effectors *Nqo1* and *Ho-1* in the kidney, further contributing to the antioxidant effects observed. We also observed that Keap1 immunostaining was reduced in the kidneys after HMC treatment, which is also consistent with the notion that Nrf2 signaling was enhanced by this flavonoid [26]. The inhibition of NFκB by HMC is possibly an additional mechanism for containing oxidative stress in SDCF-induced AKI, as this pro-inflammatory transcription factor is redox sensitive [1,9,26,34]. 

Besides being a potent inducer of antioxidant responses, Nrf2 can also contribute to reducing inflammation [26,56]. This concept is supported by several preclinical studies evaluating Nrf2 during the modulation of inflammatory states. For instance, Nrf2 activity may reduce the expression of pro-inflammatory cytokines (including tumor necrosis factor-α (TNF-α) and IL-6) in immune cells, such as neutrophils and macrophages. High Nrf2 expression counteracts the expression of pro-inflammatory genes by inhibiting NFκB and Nrf2 disruption aggravates the inflammatory response in models of sepsis, pleurisy, emphysema, and autoimmune diseases [56]. Through GATA binding protein-3 (GATA-3) induction, Nrf2 can simultaneously suppress the production of IFN-γ and increase the production of Th2 cytokines IL-4, IL-5, and IL-13 [57] and CD4^+^ T cells from Nrf2 knockout mice produce more IFN-γ and less Th2 cytokines [57]. Finally, Nrf2 can promote the production of IL-10 and transforming growth factor-β (TGF-β) in FoxP3-expressing Treg cells [56]. Thus, this robust body of evidence indicates Nrf2 per se is crucial for the control of inflammation.

## 5. Conclusions

Although considered a drug of first choice for many clinical conditions related to pain and inflammation, SDCF may induce kidney toxicity. One relevant pathological mechanism of SDCF for the induction of renal damage involves the depletion of antioxidant defenses together with increased oxidative stress. Therefore, alternative pharmacological tools with antioxidant properties and no adverse reactions for renal tissue need to be validated to reduce the potential negative impacts of this condition. Data obtained from this study indicates HMC improves antioxidant status, as measured by total antioxidant capacity in blood and renal tissue, and GSH levels in the kidney. Reduced lipid peroxidation in kidney and blood was also observed after HMC treatment. The alleviation of SDCF-induced nephrotoxicity by HMC was not limited to redox state modulation since it also inhibited pro-inflammatory cytokines in blood and kidney and increased production of the anti-inflammatory cytokine IL-10 in the kidney. These antioxidant and anti-inflammatory properties of HMC in the present model reduced the damage in renal tissue caused by SDCF with a contribution from the activation of the Nrf2/ARE redox-sensitive pathway and a reduction in Keap1. Thus, the present study supports clinical investigation of HMC as an effective therapeutic option for the treatment of SDCF-induced AKI.

## Data Availability

Data is contained within the article.
